# Role of Radical Prostatectomy in Oligo-Metastatic Hormone-Sensitive Prostate Cancer: A Systematic Review and Meta-Analysis

**DOI:** 10.3390/cancers17172757

**Published:** 2025-08-24

**Authors:** Karthik Rajan, Kalpesh Parmar, Shri-Ishvarya Rajamoorthy, Robert Geraghty, Eleanor Whyte, Bhavan Prasad Rai

**Affiliations:** 1 Department of Urology, Freeman Hospital, Newcastle upon Tyne NE7 7DN, UK; 2 Department of Urology, Morriston Hospital, Swansea SA6 6NL, UK

**Keywords:** metastatic prostate cancer, oligo-metastatic prostate cancer, radical prostatectomy, cytoreductive surgery, androgen deprivation treatment, systemic anti-cancer treatment

## Abstract

Introduction and Aims: Androgen deprivation therapy (ADT) with systemic anti-cancer treatment (SACT) ± palliative radiotherapy (pRT) is the current standard of care for Oligo-metastatic hormone-sensitive prostate cancer (o-mHSPC). Cytoreductive radical prostatectomy (cRP) has gained interest in this group of patients, with potential benefits including reduced tumour burden and a lower risk of local events from disease progression. In this review, we compare both survival outcomes and local event rates between cRP and upfront ADT ± SACT. Methods: All randomised trials and observational studies comparing cRP with standard treatment (ST), which we defined as ADT ± SACT for o-mHSPC, were included in the review. The study protocol was registered in PROSPERO (CRD42024516586), and the review was conducted in accordance with the Preferred Reporting Items for Systematic Reviews and Meta-Analyses (PRISMA). The databases searched included Embase, Medline, Cochrane Library, PubMed, and Web of Science. A risk of bias assessment was performed for the included studies as recommended by the Cochrane Handbook of Systematic Reviews and Interventions. The primary outcome measures were Overall Survival (OS), Cancer-Specific Survival (CSS), Progression-free Survival (PFS), Castrate-resistant Prostate Cancer-free Survival (CRPC-FS), and local complication rates. The secondary outcome measures were complication rates and functional outcomes post-cRP. Results: A total of 5130 studies were identified for this review (5119 by database searching and 11 through manual searching). Eight studies were included in the review, comprising 611 patients. cRP was identified to have superior OS (HR: 0.56 (95% CI: 0.34–0.92), I^2^ = 0%, *p* = 0.02 (very low certainty)) and CSS (HR: 0.27 (95% CI: 0.15–0.47), I^2^ = 0%, *p* < 0.0001 (very low certainty)). The PFS (HR: 0.67 (95% CI: 0.34–1.33), I^2^ = 58%, *p* = 0.25 (very low certainty)) and CRPC-FS (HR: 0.67 (95% CI: 0.32–1.43), I^2^ = 57%, *p* = 0.30 (very low certainty)) were similar between the two groups. The rates of local events were significantly lower in patients undergoing cRP (RR 0.27 (95% CI: 0.13–0.59), I^2^ = 17%, *p* = 0.001 (low certainty)). The rates of Clavien–Dindo (CD) grade 3 or higher complications ranged from 0% to 13.1%. Additionally, the reported continence rates ranged from 81.5% to 91.3%. The review is limited by the lack of a uniform definition for o-mHSPC and the predominance of low-quality, heterogeneous studies. Despite mitigation strategies, the overall certainty of evidence remains very low per GRADE assessment. Conclusion: cRP significantly reduces local event rates compared with ST and offers comparable PFS and CFPC-FS, with superior OS and CSS in the cRP arm compared to the ST arm in patients with o-mHSPC. However, there is a paucity of high-quality literature on this subject. Ongoing randomised controlled trials may soon clarify the role of cRP in the context of o-mHSPC concerning survival benefits.

## Simple Summary

Oligometastatic prostate cancer is a form of prostate cancer that has spread beyond the prostate, but only to a few areas. Standard treatment includes hormone therapy, sometimes combined with other cancer drugs or radiotherapy. In the past, surgery to remove the prostate (radical prostatectomy) was only offered when cancer was confined to the prostate. However, we now know that surgery can also be safely performed in carefully selected patients with limited spread. This review compares surgery with hormone treatment ± additional drugs in terms of survival and effect on local symptoms. It found that surgery provided better control of local symptoms and improved both overall and cancer-specific survival. The time until cancer progression or development of resistance to hormone therapy was similar between the two groups. While these results are encouraging and support surgery as a treatment option, the available evidence remains weak, and results from more high-quality research are expected soon to provide more clarity.

## 1. Introduction

The management of metastatic hormone-sensitive prostate cancer (mHSPC) has evolved significantly over the last decade. Traditional management of these patients involved androgen deprivation therapy (ADT) with a median survival of about 50 months with this strategy alone [1]. However, metastatic prostate cancer represents a heterogeneous cohort of patients with varying survival outcomes based on multiple predictive factors. Among the most important are the volume of metastatic disease, as identified by the CHAARTED and STAMPEDE trials [2,3]. These studies led to the stratification of metastatic prostate cancer into high- and low-volume categories based on the number of metastases and the presence or absence of visceral metastasis. However, there is still a lack of consensus in defining oligo-metastatic hormone-sensitive prostate cancer (o-mHSPC), and varying descriptions are reported in the literature [4].

o-mHSPC is thought to be a state of transition on the pathway of cancer progression towards widespread metastatic disease. It is believed to potentially have a more favourable prognosis [5]. o-mHSPC thus signifies a subset of patients for whom, theoretically, aggressive multimodal treatment can achieve better survival outcomes. The concept of cytoreductive treatment of the primary organ in the context of metastatic malignancy has been investigated in renal and ovarian cancers, with survival benefits identified in a select cohort of patients [6,7]. Although the exact biological mechanism for this is unknown, it is highlighted that this could be the result of reducing the overall tumour burden and interrupting the metastatic cascade, as the primary tumour may act as a continuous source of metastatic seeding [5,8]. Furthermore, local progression of the tumour results in genitourinary symptoms such as bladder outlet obstruction or ureteric obstruction, the management of which can be challenging and consequently lead to a significant adverse impact on quality of life [9]. Cytoreductive radical prostatectomy (cRP) targeting the primary tumour aims to address both issues—first by interrupting the ‘self-seeding’ mechanism to slow disease progression, and second, through the prevention of local progression [8].

The STOPCAP systematic review reported that prostate radiotherapy (RT) combined with ADT resulted in a 7% improvement in 3-year survival in patients with fewer than five bony metastases [10]. Additionally, the PEACE-1 trial evaluating the addition of RT to ADT in combination with androgen receptor pathway inhibitors (ARPI) reported significant improvement in progression-free survival (PFS) from 4.4 to 7.5 years in patients with low-metastatic burden [11]. Based on these data, there has been a paradigm shift in management strategy for patients with o-mHSPC from ADT alone to aggressive multimodal therapy as the current standard of care (SoC), including the addition of RT (72 Gy in 2 Gy fractions or equivalent) to ADT or ADT + ARPI [12].

Cytoreductive radical prostatectomy as an alternative for local control as part of multimodality management of o-mHSPC has been gaining increasing interest over the past decade, with retrospective studies and institutional series reporting encouraging results in terms of oncological outcomes, local control, and feasibility of surgery [13,14,15].

This systematic review aims to comprehensively evaluate the available evidence comparing oncological outcomes and local events of cRP, as part of a multimodal treatment strategy, with standard treatment (ST), defined in this review as ADT ± upfront systemic anti-cancer therapy (SACT), including ARPI and/or docetaxel in patients with o-mHSPC. Additionally, the review evaluates the toxicity and functional outcomes associated with cRP in this patient population.

## 2. Methodology

### 2.1. Search Strategy

The study protocol for this review was registered with PROSPERO on 26/02/2024 (CRD42024516586). The systematic review was performed in accordance with the Cochrane guidelines and the Preferred Reporting Items for Systematic Reviews and Meta-Analyses (PRISMA) [16]. The databases searched included Embase, Medline, Cochrane Library, PubMed, and Web of Science. The search was conducted in January 2025. The search terms used for the strategies are included in Supplementary File S1.

### 2.2. Inclusion Criteria and Data Extraction

Randomised control trials, prospective and retrospective observational studies, comparing cytoreductive radical prostatectomy as part of a multimodality approach with standard treatment (ADT ± SACT (ARPI and/or Docetaxel)) were eligible for inclusion. Only adult participants diagnosed with oligo-metastatic prostate cancer, defined as five or fewer extra-pelvic nodal or skeletal metastases, were included in the review.

Studies were assessed by two reviewers independently (KR, KP) to determine their suitability to be included in this review, according to the inclusion criteria. Data from the included studies were independently extracted by the two reviewers (KR, KP) and verified. Any disagreements for study eligibility or data extraction were resolved by consensus and review by the senior author (BR).

### 2.3. Outcome Measures

#### 2.3.1. Primary Outcomes

- Progression-free survival (PFS)
- Cancer-specific survival (CSS)
- Overall survival (OS)
- Local event Rates/Local event free survival (LEFS)
- Castrate-Resistant prostate cancer-free survival (CRPC-FS)

#### 2.3.2. Secondary Outcomes

- Complication rates
- Functional outcomes

### 2.4. Assessment of Evidence Quality and Outcome Certainty

A risk of bias assessment was performed by 2 reviewers (KR, BR) for the included studies as recommended by the Cochrane Handbook of Systematic Reviews and Interventions [17]. The risk of bias in non-randomised comparative studies will be assessed as above, along with an additional item to evaluate the risk of findings being explained by confounding. The potential confounding factors are:

- Number of Metastatic Sites
- Location of Metastatic Sites
- Age
- Performance Status
- Type of imaging used to define Metastasis (Molecular imaging vs. Conventional Cross-sectional imaging, e.g., CT/MRI/Bone Scan).

The certainty of evidence for each outcome was evaluated using the Grading of Recommendations Assessment, Development and Evaluation (GRADE) approach [18].

### 2.5. Data Synthesis

Demographic data and event rates were described narratively. A narrative synthesis was adopted when study cohorts were judged to be at high risk of bias (RoB) for more than one prespecified confounder or when outcome data were presented in a format not suitable for meta-analysis. Meta-analyses were performed when no more than one of the five prespecified confounders was at high RoB, and data were presented in Forest plots. Given the anticipated clinical and methodological heterogeneity, a random-effects model was used to calculate pooled estimates of treatment effect and corresponding 95% confidence intervals (CIs).

For time-to-event outcomes, log hazard ratios and their variances were combined using the generic inverse variance method. For dichotomous outcomes, the Mantel–Haenszel method was applied, and results were reported as risk ratios (RRs) with 95% CIs. A *p*-value of <0.05 was considered statistically significant. Statistical heterogeneity was assessed using the chi-squared (χ^2^) test on N − 1 degrees of freedom (α = 0.1) and quantified using the I^2^ statistic, with thresholds of 25%, 50%, and 75% indicating low, moderate, and high heterogeneity, respectively. Data analysis was performed using Review Manager (RevMan) version 5.4.

## 3. Results

**Figure 1:**
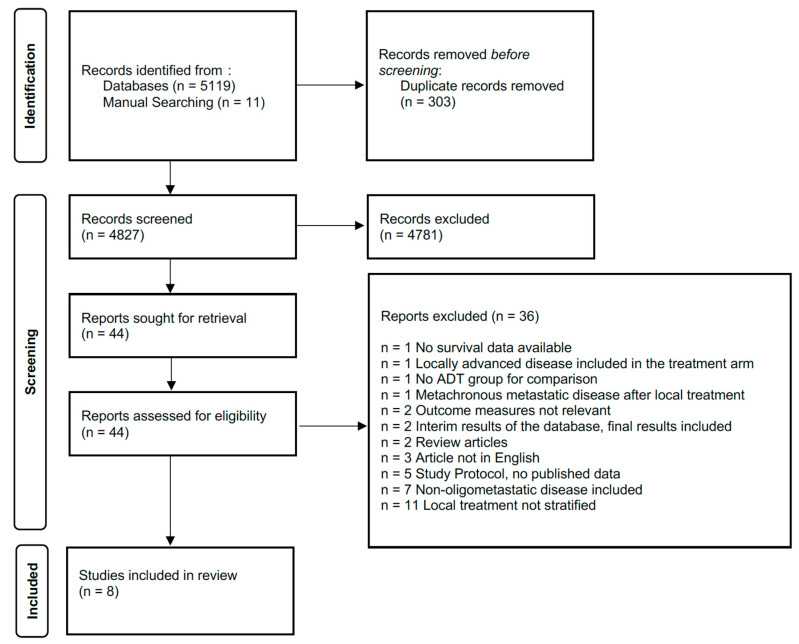
PRISMA Chart.

The PRISMA flow diagram for study selection is depicted in Figure 1. A total of 5119 studies were identified through database searching, and 11 additional studies were identified through manual searching. Subsequently, 44 studies were identified that required evaluation of the full text. Of these, 8 studies were identified that fulfilled the inclusion criteria and were incorporated into the review [13,19,20,21,22,23,24,25]. Three studies were conducted prospectively [21,22,24]. Six studies were single-centre studies, while two studies reported multicentre data; notably, Lumen et al. reported outcomes from a prospective registry of patients with newly diagnosed metastatic prostate cancer [21,24]. Chi et al. performed a prospective propensity score–matched pair analysis, whereas Heidenreich et al. conducted a retrospective case-control matched analysis [13,22] (Table 1).

The review included 611 patients, with 272 patients allocated to the cRP arm (cRP + ADT ± SACT ± adjuvant radiotherapy) and 339 allocated to the ST arm (ADT ± SACT). Treatment received and demographic/tumour data are shown in Table 2 and Table 3, respectively.

Definitions for o-mHSPC varied across studies, with all using a threshold of 3–5 metastatic lesions. All studies excluded patients with unresectable disease or visceral metastases. Metastatic disease was primarily identified using conventional imaging in all studies [13,19,20,21,22,23,24,25]. Two studies selectively employed additional molecular imaging with Choline-PET [19] or PSMA-PET [22], based on clinical judgement.

Two studies performed cRP using a robotic approach [19,25]; one study reported both open and robotic approaches [21]; one study used an open approach [13]; and the remaining studies did not specify the surgical technique [20,22,23,24]. An extended template pelvic lymph node dissection (PLND) was performed in all six studies [13,19,20,21,23,25]. One study did not specify the extent of pelvic lymph node dissection (PLND), while another study neglected to indicate whether a PLND was performed and, if so, its extent [22,24] (Table 2).

Median follow-up for the cRP arm ranged from 30 to 64.2 months, and for the ST arm from 29 to 82.2 months. Adjuvant radiotherapy following cRP was reported in two studies [13,19]. This included 4 patients (14.3%) in the cRP arm in Heidenreich et al., who had positive surgical margins and 12 patients (30%) in Mistretta et al., who had either N1 disease or T3 disease with positive margins [13,19].

Upfront SACT was administered in the form of Abiraterone for all patients in both arms in one study [20]. Both Abiraterone and docetaxel were administered as upfront SACT in Lumen et al. [21]. Abiraterone was administered to four patients (8.3%) in the cRP arm and six patients (17.1%) in the ST arm, while upfront Docetaxel was given to seven patients (14.6%) undergoing cRP compared to six patients (17.1%) receiving ST [21] (Table 2).

### 3.1. Primary Outcomes

#### 3.1.1. Progression-Free Survival (PFS)

**Figure 2:**
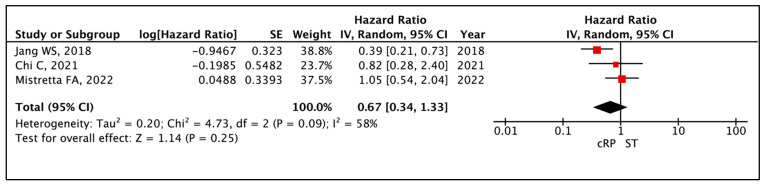
Primary Outcome: Time to event analysis—Progression Free Survival [19,22,25].

The median PFS reported across all included studies ranged from 30.6 to 75 months. Meta-analysis of appropriate hazard ratios (HRs) from three comparative studies [19,22,25] demonstrated no significant difference in PFS between the two cohorts (HR: 0.67 (95% CI: 0.34–1.33), I^2^ = 58%, *p* = 0.25 (very low certainty)) (Figure 2).

#### 3.1.2. Cancer-Specific Survival (CSS)

**Figure 3:**
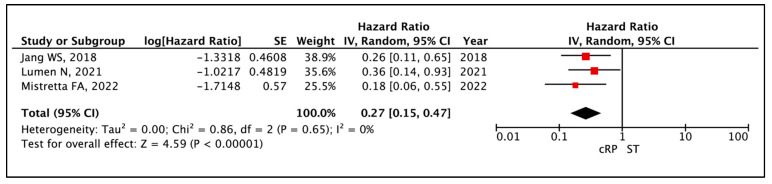
Primary Outcome: Time to event analysis—Cancer-Specific Survival [19,21,25].

Heidenreich et al. reported a median CSS of 47 months in patients who underwent a cRP. In two other studies, the median CSS was not reached [23,25]. In the ST arm, the median CSS ranged from 40 to 40.5 months. The meta-analysis of appropriate HRs from three comparative studies [19,21,25] showed a prolonged CSS when patients underwent a cRP (HR: 0.27 (95% CI: 0.15–0.47), I^2^ = 0%, *p* < 0.0001 (very low certainty)) (Figure 3). Lan et al. reported that both 3- and 5-year CSS outcomes were similar between the cRP and ST cohorts [23] (Table 4).

#### 3.1.3. Overall Survival (OS)

**Figure 4:**
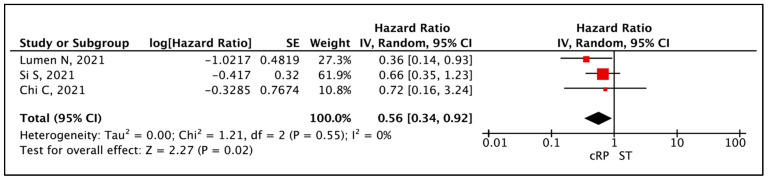
Primary Outcome: Time to event analysis—Overall Survival [20,21,22].

The meta-analysis of appropriate HRs from three comparative studies [20,21,22] showed that patients who underwent a cRP had a prolonged OS compared to ST (HR: 0.56 (95% CI: 0.34–0.92), I^2^ = 0%, *p* = 0.02 (very low certainty)) (Figure 4). Three additional studies have reported on OS, not suitable for inclusion in the pooled analysis [13,21,24]. Heidenreich et al. (91.3% vs. 78.9, *p* = 0.048) and Lumen et al. (2-yr OS 93% vs. 69%, *p* = 0.007) report OS outcomes in favour of cRP compared to ST [13,21]. Steuber et al. did not report any significant difference in OS between the two groups (*p* = 0.25) [24] (Table 4).

#### 3.1.4. Castrate Resistant Prostate Cancer-Free Survival (CRPC-FS)

**Figure 5:**
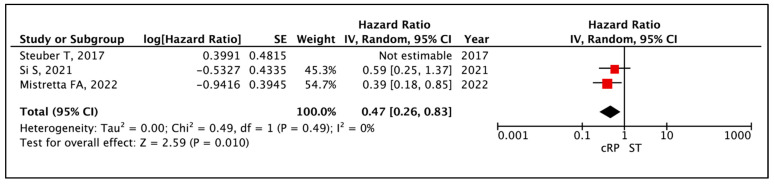
Primary Outcome: Time to event analysis—Castrate Resistant Prostate Cancer-Free Survival [19,20,24].

The meta-analysis of appropriate HRs available in three comparative studies showed progression to CRPC was similar between cRP and ST (HR: 0.67 (95% CI: 0.32–1.43), I^2^ = 57%, *p* = 0.30 (very low certainty)) [19,20,24] (Figure 5). Two other studies reported on CRPC-FS, with Lan et al. reporting no significant difference between cRP and ST (*p* = 0.118) and Heidenreich et al. reporting cRP to be superior to ST (*p* = 0.014), with median CRPC-FS in studies ranging from 35 to 40 months in the cRP arm as compared to 21 to 20 months in the ST arm across both studies [13,23].

#### 3.1.5. Local Events

**Figure 6:**
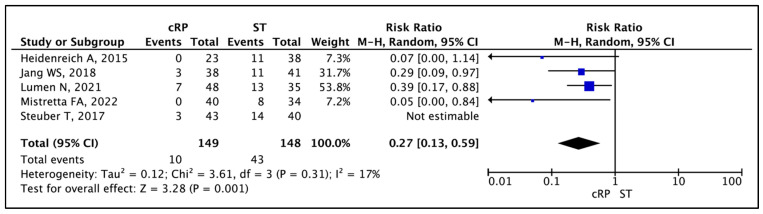
Local Events [13,19,21,24,25].

Across five comparative studies, local event rates ranged from 0% to 14.6% in the cRP arm, compared with 23.5% to 37.1% in the ST arm [13,19,21,24,25]. All individual studies reported a significantly lower event rate in patients who underwent a cRP when compared to ST. The meta-analysis of appropriate RRs available in four comparative studies showed significantly lower risk of local event in patients who underwent cRP (RR 0.27 (95% CI: 0.13–0.59), I^2^ = 17%, *p* = 0.001 (low certainty)) [13,19,21,25] (Figure 6). Lumen et al. reported a prolonged 2-year local outcomes when patients underwent a cRP (92 ± 4% vs. 60 ± 9%, HR 0.25, 95% CI 0.10–0.64; *p* = 0.004) [21].

### 3.2. Secondary Outcomes—Post cRP

#### 3.2.1. Complications

Five studies reported on overall complication rates following cRP [13,20,21,22,25], ranging from 14.6% to 39.1% (Table 5). Major complication rates (Clavien–Dindo ≥ 3), reported in three studies, ranged between 0% to 13.1% [13,20,25].

#### 3.2.2. Functional Outcomes

Two studies reported on functional outcomes in terms of continence post cRP [13,20]. Heidenreich et al. reported that 91.3% of patients were continent, requiring ≤ 1 pad per day [13]. Si et al. reported continence rates of 81.5% with no information on pad use [20]. Neither study reported the time frame of assessment of continence. In addition, Steuber et al. reported two cases of anastomotic stricture and one patient with severe incontinence, all requiring further intervention [24].

### 3.3. Quality of Evidence and Certainty of Outcomes

**Figure 7:**
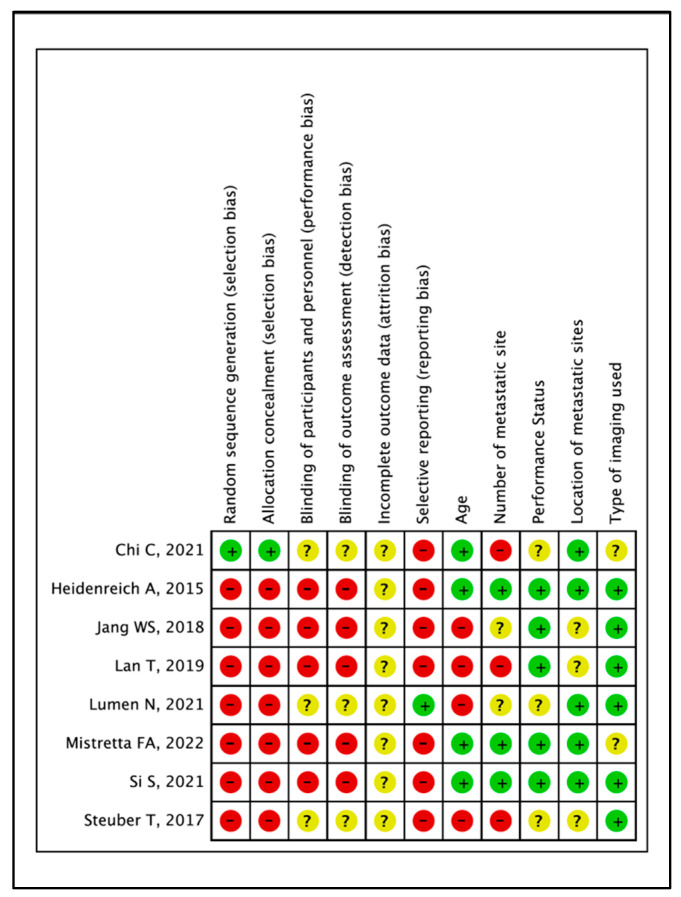
Risk of Bias Summary [13,19,20,21,22,23,24,25].

One study was judged to have a low risk of selection bias as it was prospective and propensity score matched [22]. All the other studies were judged to have a high risk of selection bias [13,19,20,21,23,24,25]. Five studies were judged to have a high risk of performance and detection bias as they were retrospective [13,19,20,23,25]. Three prospective studies were judged to have unclear risk as they were conducted prospectively, with no information on whether any methods were used for blinding [21,22,24]. Lumen et al. have published a protocol for their prospective registry, and were judged to have low risk, while all the other studies have a high risk of selective reporting with no prior protocol [21] (Figure 7).

The certainty of evidence for all pooled outcome measures was judged as either low or very low (Table 6). The reasons for this were due to bias, inconsistency and imprecision, reflecting clinical and/or statistical heterogeneity and limitations in the design and conduct of the included studies.

## 4. Discussion

This review indicates that cRP was consistently superior to ST in preventing local progression-related events across all included studies. However, the impact of cRP on oncological outcomes varied among studies. cRP was associated with either improved or comparable CSS and OS compared to patients who did not undergo surgery for o-mHSPC. No significant differences were observed between the cRP and ST cohorts in PFS or time to CRPC.

The natural progression of mHSPC is the eventual development of CRPC despite systemic treatment [26]. During this course, an estimated 56% of patients develop symptoms from local progression [9]. The STAMPEDE trial reported a PFS survival benefit with the addition of palliative prostate radiotherapy in patients with low-volume metastatic disease. However, radiotherapy did not improve the rates of urinary symptoms and local events in comparison to ST [2]. This outcome has also been previously reported in the context of CRPC, where patients who underwent RP showed fewer local complications compared to patients receiving ADT only (20% vs. 54.3%, *p* = 0.001) or radiotherapy (20% vs. 46.7%, *p* = 0.007) [9]. These findings, which align with the results from our review, support the role of cRP as an important intervention in select patients at high risk of future local complications, such as those with large prostate volumes for whom endoscopic bladder outlet surgery may be suboptimal, or those with locally advanced disease where ureteric obstruction may be imminent. Furthermore, cRP may be the local treatment option of choice where RT is contraindicated, for example in patients with inflammatory bowel disease.

The variation in oncological outcomes in this review is anticipated considering the diagnostic and treatment heterogeneity between the two cohorts, reflecting evolving evidence and variation in global practice. Despite these limitations in well-matched and balanced comparative studies, pooled analyses suggested a potential survival advantage of cRP in both CSS and OS. These findings are consistent with other population-based analyses and randomised control trials which did not fulfil the inclusion criteria within this review. Antwi et al. conducted a propensity-matched population-based analysis using data from the Surveillance Epidemiology and End Results (SEER) database [27]. The cohort was unselected for metastatic burden but still showed a significant OS and CSS benefit for cRP compared to patients who received only ST. Dai et al. conducted a randomised trial comparing ADT with cytoreductive treatment [28]. However, 15% of patients in the treatment arm received RT instead of cRP, rendering their findings unsuitable for inclusion in this review. Nonetheless, they reported a significant improvement in OS, which is consistent with the results of our review. The results from ongoing randomised trials comparing cRP with ADT are awaited and will be able to provide evidence of higher quality than the current literature to help address this question [29].

Jang et al. reported major complication rates of 5.3% for cRP performed robotically [25]. Similarly, data from the TRoMbone randomised feasibility trial by Sooriakumaran et al. demonstrated a major complication rate of 8.8% [30]. These findings are comparable to outcomes from RARP for locally advanced prostate cancer (3.1 to 22.6%) [31]. The functional outcomes were reported by Lan et al. and Heidenreich et al., who reported continence rates of 81.5% and 93.3%, respectively, which are comparable to outcomes reported for RARP in locally advanced disease (64–100%) [31]. Given the inconsistent and limited reporting of functional outcomes in the included studies, further high-quality randomised controlled trials with standardised definitions and uniform reporting are needed to better understand the impact of cytoreductive prostatectomy in this domain.

In light of recent advances in systemic therapies for the management of o-mHSPC, the relevance of cytoreductive treatment has been questioned [32]. However, findings from the PEACE-1 trial support the continued role of cytoreductive interventions, even in the context of ARPIs and other novel systemic agents now considered standard of care [11]. Moreover, the addition of SACT to ADT is associated with metabolic, cognitive and bone health concerns—issues particularly relevant in patients with pre-existing cardiovascular disease or osteoporosis, which may be more prevalent given the typical age demographic of this cohort [33,34,35]. In our review, the median patient age ranged from 61 to 76 years. cRP, therefore remains a potential alternative in selected patients, offering both oncological benefit through local tumour control and symptom relief from urinary obstruction. With the continued evolution of targeted therapies, the management of metastatic hormone-sensitive prostate cancer is likely to become increasingly personalised, with emerging evidence supporting multimodal approaches, including metastasis-directed treatments in selected patients, in the management of o-mHSPC [36,37]. Our review suggests that cRP is a safe and feasible intervention and should be considered as a component of the therapeutic armamentarium in the management of oligometastatic hormone-sensitive prostate cancer (o-mHSPC).

Our review has several limitations, the most significant being the lack of a standardised definition for o-mHSPC, which led to variable inclusion criteria, clinical heterogeneity, and considerable variation in treatment practices and outcome reporting. Although the initial protocol specified inclusion of studies with ≤4 metastatic lesions, we subsequently broadened this to ≤5 lesions, reflecting the contemporary definition adopted in both published and ongoing RCTs on this topic [28,38]. This has also been highlighted by other systematic reviews on this subject in the past [20,39]. There is a paucity of high-quality literature on this topic, with the majority of studies being retrospective or methodologically unbalanced prospective designs, often carrying a high or unclear risk of bias across most domains. We acknowledge that this heterogeneity impacts the pooled results. To mitigate this, we used pre-specified confounders and conducted pooled analyses only when studies were balanced for these confounders through matching or statistical modelling. Despite these measures, the certainty of the evidence remains very low, as confirmed by the GRADE approach; therefore, the results should be interpreted with caution.

## 5. Conclusions

cRP has been shown to significantly reduce local event rates in patients with o-mHSPC. It also provides PFS and time to CRPC outcomes comparable to those achieved with ADT ± SACT ± palliative RT, while demonstrating equivalent or even superior OS and CSS compared with patients managed without cRP. Although the results should be interpreted with caution and cannot be considered as firm evidence due to the very low certainty of outcomes, the results support consideration of cRP in selected patients as part of a multi-disciplinary approach.

The body of literature on this topic has evolved significantly over the past two decades. Several randomised trials are currently underway, and their forthcoming results may offer greater clarity on whether cRP confers superior oncological benefit compared to systemic therapy ± radiotherapy [29]. The most recent European Association of Urology (EAU) guidelines have incorporated radiotherapy to the primary tumour as a recommended treatment option for patients with o-mHSPC [12]. However, similar to our review, many ongoing randomised trials do not include prostate radiotherapy in their treatment arms, raising concerns about the applicability of their findings in the context of a rapidly evolving therapeutic landscape.

## Figures and Tables

**Table 1:** Characteristics of included studies.

Authors	Outcomes Compared	Definition of o-mHSPC	Study Design	Centres	Study Characteristics	Site of Study	Study Period
Heidenreich 2015 [13]	cRP vs. ST	≤3 bony mets, No extra pelvic LN, No bulky (>3 cm pelvic LN)	Retrospective	Single	Case Control—Matched	Germany	Not mentioned
Steuber 2017 [24]	cRP vs. ST	1–3 bony mets, no visceral mets, ≤T3	Prospective	Multicentre (3)	Case Control	Germany, Denmark	2008 to 2015
Jang 2018 [25]	cRP vs. ST	≤5 bony mets, No visceral mets	Retrospective	Single	Observational Cohort	South Korea	2005 to 2015
Lan 2019 [23]	cRP vs. ST	≤5 bony mets, No visceral mets	Retrospective	Single	Observational Cohort-Consecutive	Europe	2005 to 2016
Chi 2021 [22]	cRP vs. ST vs. ADT + Chemo + cRP	<5 metastatic lesions—extra pelvic nodes/bones	Prospective	Single	Propensity score matched	China	2014 to 2019
Lumen 2021 [21]	cRP vs. RT vs. ST	<4 bony mets, no visceral mets	Prospective	Multicentre (5)	Observational Cohort	Belgium	2014 to 2020
Si 2021 [20]	cRP vs. ST	≤5 bony mets, No visceral mets	Retrospective	Single	Observational Cohort	China	2010 to 2015
Mistretta 2022 [19]	cRP + RT vs. ST	≤5 bony mets, No visceral mets, locally resectable (T1–T3)	Retrospective	Single	Observational Cohort	Italy	2010 to 2018

RP = Radical Prostatectomy, ST = Standard treatment, ADT = Androgen deprivation treatment.

**Table 2:** Treatment characteristics of included studies.

Authors	Groups	cRP (*n*)	ST (*n*)	Upfront Rx in cRP Arm	Upfront in ST Arm	Imaging Modality
Heidenreich 2015 [13]	cRP vs. ST	23	38	Open RP + ePLND + Adjuvant RT if positive margins (66.6 Gy)	ADT only	Conventional imaging only
Steuber 2017 [24]	cRP vs. ST	43	40	RP ^a,b^ + ADT	ADT only	Conventional imaging only
Jang 2018 [25]	cRP vs. ST	38	41	RARP + ePLND + ADT	ADT only	Conventional imaging only
Lan 2019 [23]	cRP vs. ST	35	76	RP ^a^ + ePLND + ADT	ADT only	Conventional imaging only
Chi 2021 [22]	cRP vs. ST	18	18	RP ^a^ + PLND ^c^ + ADT	ADT only	Conventional + FDG/PSMA-PET
Lumen 2021 [21]	cRP vs. ST	48	35	RP (Open + RARP) + ePLND ± ST ± SACT (Docetaxel/Abiraterone)	ADT ± SACT (Docetaxel/Abiraterone)	Conventional imaging only
Si 2021 [20]	cRP vs. ST	27	57	RP ^a^ + ePLND + ADT + Abiraterone	ADT + Abiraterone	Conventional imaging only
Mistretta 2022 [19]	cRP vs. ST	40	34	RARP + ePLND + ADT ± Adjuvant RT	ADT only	Conventional + Choline PET

cRP = Cytoreductive radical prostatectomy, ST = Standard treatment, RP = Radical Prostatectomy, RT = Radiotherapy, ADT = Androgen deprivation treatment, RARP = Robot-assisted radical prostatectomy, ePLND = Extended pelvic lymph node dissection, PLND = Pelvic lymph node dissection. ^a^ Not specified if open or robotic radical prostatectomy was performed. ^b^ Did not specify if lymphadenectomy was performed or the extent. ^c^ Extent of lymphadenectomy not described.

**Table 3:** Demographics of included studies.

Author	Age (Years)	*p*-Value	PSA (ng/mL)	*p*-Value	Gleason Score Pre-op (*n*,%)	*p*-Value	Metastasis Characteristic (*n*, %)	*p*-Value	No. of Mets	*p*-Value
Heidenreich 2015 [13]	**cRP** 61 (42–69) **ST** 64 (47–83) (Mean, Range)	NR	**cRP** 135.2 (3.5–150.4) **ST** 105 (45–195) (Mean, range)	0.049	**cRP** ≤7 = 5 (21.7%) 8 = 7 (30.4%) 9 = 7 (30.4%) 10 = 4 (17.4%) **ST** ≤7 = 11 (28.9%) 8 = 11 (28.9%) 9 = 8 (21.1%) 10 = 4 (10.5%)	NR	**cRP** - M1a = 3 (13.1%) - M1b = 23 (100%) **ST** - M1a = 4 (10.5%) - M1b = 38 (100%)	NR	**cRP** 2.1 (1–3) **ST** 2.5 (1–5) (Mean, Range)	NR
Steuber 2017 [24]	**cRP** 70 **ST** 65 (Median)	<0.01	**cRP** 42.5 **ST** 29 (Median)	0.02	**cRP** ≤7 = 15% 8 = 32.5% 9 = 40% 10 = 12.5% **ST** ≤7 = 30.2% 8 = 30.2% 9 = 34.9% 10 = 4.7%	0.22	NR	NR	NR	NR
Jang 2018 [25]	**cRP** 65 (62–69) **ST** 71 (67–76) (Median, IQR)	<0.001	**cRP** 39.0 (15.0–84.5) **ST** 50.0 (23.8–162.8) (Median, IQR)	0.206	**cRP** ≤8 = 26 (68.4%) ≥9 = 12 (31.6%) **ST** ≤8 = 24 (58.5%) ≥9 = 17 (41.5%)	0.484	NR	NR	NR	NR
Lan 2019 [23]	**cRP** 67.83 ± 7.19 **ST** 71.7 ± 7.73 (Mean, SD)	0.030	**cRP** 90.4 ± 152.8 **ST** 502.9 ± 806.0 (Median, SD)	0.003	**cRP** ≤7 = 27 (77.1%) 8 = 6 (17.1%) 9–10 = 2 (5.7%) **ST** ≤7 = 27 (35.5%) 8 = 27 (35.5%) 9–10 = 21 (27.6%)	0.001	NR	NR	**cRP** 2.37 ± 1.22 ST 2.93 ± 1.12 (Mean, SD)	0.019
Chi 2021 [22]	**cRP** 68.05 ± 8.24 **ST** 68.22 ± 6.67 (Mean, SD)	0.94	**cRP** 97.43 (124.61) **ST** 78.25 (58.95) (Median, IQR)	0.99	**cRP** 7 = 6 (33.33%) 8 = 8 (44.44%) 9 = 4 (22.22%) **ST** 7 = 3 (16.67%) 8 = 9 (50.0%) 9 = 6 (33.33%)	0.57	**cRP** - M1a = 4 (22.2%) - M1b = 16 (88.9%) **ST** - M1a = 4 (22.2%) - M1b = 15 (83.3%)	*p* (M1a) = 0.15 *p* (M1b) = 0.032	NR	NR
Lumen 2021 [21]	**cRP** 64 (59–72) **ST** 74 (69–84) (Median, IQR)	<0.001	**cRP** 19 (11–42) **ST** 47 (17–156) (Median, IQR)	0.008	**cRP** ≤7 = 9 (20.9%) 8 = 10 (20.8%) 9–10 = 28 (58.3%) **ST** ≤7 = 8 (25.1%) 8 = 11 (34.4%) 9–10 = 13 (40.6%)	0.318	**cRP** - M1a = 23 (47.9%) - M1b = 25 (52.1%) **ST** - M1a = 10 (28.6%) - M1b = 25 (71.4%)	0.112	NR	NR
Si 2021 [20]	**cRP** 76.67 ± 9.66 **ST** 76.42 ± 9.69 (Mean, SD)	0.914	**cRP** 28.93 **ST** 70.83 (Median)	0.121	NR	NR	**cRP** - M1a = 5 (18.5%) - M1b = 100% **ST** - M1a = 8 (14.0%) - M1b = 100%	*p* (M1a) = 0.394 *p* (M1b) = 0.258	**cRP** 2.07 ± 0.917 **ST** 2.18 ± 1.07	0.837
Mistretta 2022 [19]	**cRP** 67 (58–68) **ST** 64 (60–74) (Median, IQR)	0.2	**cRP** 14 (9–29) **ST** 87 (35–186) (Median, IQR)	<0.001	**cRP** ≤7 = 18 (45%) 8–10 = 22 (55%) **ST** ≤7 = 9 (26.5%) 8–10 = 23 (67.6%)	0.1	**cRP** - M1a = 16 (40.0%) - M1b = 24 (60.0%) **ST** - M1a = 7 (20.6%) - M1b = 27 (79.4%)	0.1	NR	NR

cRP = Cytoreductive radical prostatectomy, ST = Standard treatment, NR = Not reported, SD = Standard deviation, IQR = Interquartile range.

**Table 4:** Primary Outcomes of included studies.

Author	Follow-Up (Months)	Primary Outcomes
Heidenreich 2015 [13]	cRP vs. ST 40.6 (3–71) vs. 44 (24–96); *p* = NS (Median, Range)	**cRP vs. ST** CSS—9 5.6% vs. 84.2%, (*p* = 0.043) OS—91.3% vs. 78.9, (*p* = 0.048) Local events—0% vs. 28.9%
Steuber 2017 [24]	cRP vs. ST 32.7 (23.5–84.6) vs. 82.2 (37.1–121.2) (Median)	**cRP vs. ST** OS—*p* = 0.25 CRPC-FS—*p* = 0.92 Local events—7% vs. 35% Multivariate analysis * HR (95%CI), *p*-value CRPC-FS—1.49 (0.58–3.83); *p* = 0.408 ^*^ Multivariate Cox proportional hazard analyses were used to reveal the predictors of survival outcomes using the following covariates: age, PSA, Biopsy Gleason Score, T stage, Number of metastases
Jang 2018 [25]	Overall cohort 40 (28–58) (Median, IQR)	**cRP vs. ST** Median PFS—75 months vs. 28 months, (*p* = 0.008) Median CSS—Not reached vs. 40 months, (*p* = 0.002) Local events—7.9% vs. 26.8% Multivariate analysis * HR (95%CI), *p*-value PFS—0.388 (0.206 0.731); *p* = 0.003 CSS—0.264 (0.107 0.650); *p* = 0.004 ^*^ Multivariate Cox proportional hazard analyses were used to reveal the predictors of survival outcomes using the following covariates: age, PSA, Biopsy Gleason Score, Charlson’s comorbidity index, T stage, N stage
Lan 2019 [23]	cRP vs. ST 35 (22–51) vs. 35 (25–45); *p* = 0.135 (Median, IQR)	**cRP vs. ST** Median PFS—32 months vs. 17 months, (*p* = 0.184) Median CRPC-FS—35 months vs. 21 months, (*p* = 0.118) Median CSS—Not reached 3-year/5-year CSS—90.8% vs. 87.9%/63.6% vs. 74.9%, (*p* = 0.773) 3-year/5-year CRPC-FS—42.7% vs. 27%/19% vs. 21%
Chi 2021 [22] Propensity Matched (Age, Gleason score, clinical TNM staging and PSA level)	cRP vs. ST 30 vs. 29 (Median)	**cRP vs. ST** Median PFS—30.6 months vs. 16.1 months, (*p* = 0.57) Multivariate analysis * HR (95%CI), *p*-value PFS—0.82 (0.28–2.37); *p* = 0.70 ^*^ Multivariate Cox proportional hazard analyses were used to reveal the predictors of survival outcomes using the following covariates: Age, T stage, N stage, M stage, Gleason Score
Lumen 2021 [21]	Overall cohort 32 (16–49) (Median, IQR)	**cRP vs. ST** 2-yr OS 93 ± 4% vs. 69 ± 9% (HR 0.28, 95% CI 0.11–0.71; *p* = 0.007) 2-yr CSS 93 ± 4% vs. 75 ± 8% (HR 0.36, 95% CI 0.14–0.94; *p* = 0.037) 2-yr LEFS 92 ± 4% vs. 60 ± 9% (HR 0.25, 95% CI 0.10–0.64; *p* = 0.004) Local events—14.6% vs. 37.1% Multivariate analysis * HR (95%CI), *p*-value OS—0.36, (0.14–0.94); *p* = 0.037 ^*^ Multivariate Cox proportional hazard analyses were used to reveal the predictors of survival outcomes using the following covariates: Age, PSA, Tumour grade, ECOG status, T stage, N stage, M stage, Additional systemic treatment
Si 2021 [20]	cRP vs. ST 64.2 (56.4–81.6) vs. 73.0 (56.0–85.4); *p* = 0.496	**cRP vs. ST** Median OS—78.6 months vs. 80.7 months, (*p* = 0.649) 3-year/5-year OS—96.2% vs. 94.7%/76.0% vs. 74.9% (*p* = NS) Mean CRPC-FS—91.86 vs. 85.07, (*p* = 0.183) Univariate analysis * HR (95%CI), *p*-value OS—0.659 (0.352–1.233); *p* = 0.192 CRPC-FS—0.587 (0.251–1.376); *p* = 0.220 ^*^ Univariate Cox proportional hazard analyses were used to reveal the predictors of survival outcomes using the following covariates: BMI, Charlson’s co-morbidity index, Smoking, Number of metastases, Gleason score, Pre-op and Post-op PSA
Mistretta 2022 [19]	cRP vs. ST 55 vs. 50; *p* = 0.8 (Median)	**cRP vs. ST** CSM—5.9% vs. 37.1%, (*p* = 0.02) Disease Progression—83.1 vs. 62.5%, (*p* = 0.8) Progression to CRPC—24.0 vs. 62.5%, (*p* < 0.01) Local events—0% vs. 23.5% Multivariate analysis * HR (95%CI), *p*-value CRPC-FS—0.40 (0.19–0.84); *p* = 0.02 PFS—1.19 (0.62–2.28); *p* = 0.6 CSS—0.18 (0.05–0.56); *p* = 0.0026 ^*^ Multivariate Cox proportional hazard analyses were used to reveal the predictors of survival outcomes using the following covariates: PSA and site of metastases

cRP = Cytoreductive radical prostatectomy, ST = Standard treatment, NS = Not specified, HR = Hazard Ratio, CSS = Cancer-specific survival, OS = Overall survival, CRPC-FS = Castrate-resistant prostate cancer free survival, PFS = Progression-free survival, LEFS = Local event free survival, CRPC—Castrate-resistant prostate cancer, CSM = Cancer-specific mortality.

**Table 5:** Outcomes of Radical Prostatectomy of included studies.

Author	Op Time (mins)	Blood Loss (mL)	Positive Surgical Margins (*n*, %)	Total Complications (*n*,%)	CD3+ Complications (*n*, %)	Hospital Stay	Continence (%, Time)
Heidenreich 2015 [13]	127 (115–145) (Mean, range)	335 (250–600) (Mean, range)	4 (14.3%)	9 (39.1%)	3 (13.0%)	7.8 days (6–13) (Mean, range)	21 (93.3%, Time NR, 0–1 pads/day)
Steuber 2017 [24]	NR	NR	67.4%	NR	NR	NR	NR
Jang 2018 [25]	147 (135–186) (Median, IQR)	300 (200–500) (Median, IQR)	30 (78.9%)	6 (15.7%)	2 (5.3%)	5 days (4–7) (Median, IQR)	NR
Lan 2019 [23]	NR	NR	10 (28.6%)	NR	NR	NR	NR
Chi 2021 [22]	NR	NR	8 (47.06%)	22.71%	NR	5 (2) Median (IQR)	NR
Lumen 2021 [21]	NR	NR	NR	7 (14.6%)	NR	NR	NR
Si 2021 [20]	165 ± 86.99 (Mean, SD)	766 ± 361.62 (Mean, SD)	NR	7 (25.9%)	0	12.78 ± 10.0 (Mean, SD)	22 (81.5%, Time NR, pad use NR)
Mistretta 2022 [19]	NR	NR	NR	NR	NR	NR	NR

NR = Not reported, SD = Standard deviation, IQR = Interquartile range.

**Table 6:** GRADE Assessment.

Cytoreductive Radical Prostatectomy Compared to Standard Treatment for Oligometastatic Prostate Cancer					
**Patient or population:** Oligometastatic Prostate Cancer **Intervention:** Cytoreductive Radical Prostatectomy **Comparison:** Standard Treatment					
**Outcomes**	**№ of participants** **(studies)** **Follow-up**	**Certainty of the evidence** **(GRADE)**	**Relative effect** **(95% CI)**	**Anticipated absolute effects**	
				**Risk with Standard Treatment**	**Risk difference with Cytoreductive Radical Prostatectomy**
Time to Event Analysis—Progression Free Survival	(3 non-randomised studies)	⨁◯◯◯ Very low ^a,b,c^	**HR 0.67** (0.34 to 1.33)	0 per 1000	**-- per 1000** (-- to --)
Time to Event Analysis—Cancer Specific Survival	(3 non-randomised studies)	⨁◯◯◯ Very low ^a,b,c,d^	**HR 0.27** (0.15 to 0.47)	0 per 1000	**-- per 1000** (-- to --)
Time to Event Analysis—Overall Survival	(3 non-randomised studies)	⨁◯◯◯ Very low ^a,b,c,d^	**HR 0.56** (0.34 to 0.92)	0 per 1000	**-- per 1000** (-- to --)
Time to Event Analysis—Progression to CRPC	(2 non-randomised studies)	⨁◯◯◯ Very low ^a,b,c^	**HR 0.47** (0.26 to 0.83)	0 per 1000	**-- per 1000** (-- to --)
Local Events	297 (4 non-randomised studies)	⨁⨁◯◯ Low ^a,b^	**RR 0.27** (0.13 to 0.59)	291 per 1000	**212 fewer per 1000** (253 fewer to 119 fewer)
**The risk in the intervention group** (and its 95% confidence interval) is based on the assumed risk in the comparison group and the **relative effect** of the intervention (and its 95% CI). **CI:** confidence interval; **HR:** hazard ratio; **RR:** risk ratio					
**GRADE Working Group grades of evidence** **High certainty:** we are very confident that the true effect lies close to that of the estimate of the effect. **Moderate certainty:** we are moderately confident in the effect estimate: the true effect is likely to be close to the estimate of the effect, but there is a possibility that it is substantially different. **Low certainty:** our confidence in the effect estimate is limited: the true effect may be substantially different from the estimate of the effect. **Very low certainty:** we have very little confidence in the effect estimate: the true effect is likely to be substantially different from the estimate of effect.					

a. Retrospective design; b. Statistical Heterogeneity; c. Clinical Heterogeneity; d. No protocol.

## Supplementary Materials

The following supporting information can be downloaded at: https://www.mdpi.com/article/10.3390/cancers17172757/s1, Supplementary File S1 [40].
