# Supplementary material for: Role of Radical Prostatectomy in Oligo-Metastatic Hormone-Sensitive Prostate Cancer: A Systematic Review and Meta-Analysis
Source: Cancers (Basel). 2025 Aug 24;17(17):2757. doi: 10.3390/cancers17172757 (PMC12427360; doi:10.3390/cancers17172757)
Supplement: Supplementary file 1 [file cancers-17-02757-s001.zip › cancers-3800888-supplementary.pdf]

Population	Metastatic prostate cancer<br>Oligometastatic prostate cancer
Intervention	Radical prostatectomy<br>Cytoreductive prostatectomy
Comparator	ADT/Chemo/RT -
Outcome	Overall survival<br>Complications

<u>Concept 1 – Prostate cancer</u>

“Prostatic Neoplasms”[Mesh] OR
“Cancer prostate”[tiab:~2] OR
“Neoplasm prostate”[tiab:~2] OR
“Malignancy prostate” [tiab:~2] OR
“Prostate cancer” [tw] OR
“Prostatic cancer” [tw] OR
“Prostate neoplasm*”[tw] OR
“Prostatic neoplasm*”[tw] OR
“Prostate malignancy”[tw] OR
“Prostatic malignanc*”[tw] OR
“PCa”[tw]

<u>Concept 2 – Metastasis</u>

"Bone Neoplasms/secondary"[Mesh] OR
"Lymphatic Metastasis"[Mesh] OR
“Lymph node metasta*”[tw] OR
“Lymphnode metasta*”[tw] OR
“Lymphatic metasta*”[tw] OR
“Bone metasta*”[tw] OR
“Bony metasta*”[tw] OR
“Osseous metasta*”[tw] OR
“Metastasis bone” [tiab:~2] OR
“Metastases bone” [tiab:~2] OR
“Oligometasta*”[tw] OR
“Oligo-metasta*”[tw] OR
“Oligo metasta*”[tw] OR
“mPCa” [tw] OR
“metastatic prostate cancer” [tw]

<u>Concept 3 – Radical prostatectomy</u>

"Cytoreduction Surgical Procedures"[Mesh] OR
"Prostatectomy"[Mesh] OR
"Prostatic Neoplasms/surgery"[Mesh] OR
“Cytoreductive prostatectom*”[tw] OR

“Cytoreductive prostatectomy”[tiab:~2] OR
“Radical prostatectomy*”[tw] OR
“Local treatment”[tw]

Final strategy

(("Prostatic Neoplasms"[Mesh] OR "Cancer prostate"[tiab:~2] OR "Neoplasm prostate"[tiab:~2] OR "Malignancy prostate" [tiab:~2] OR "Prostate cancer" [tw] OR "Prostatic cancer" [tw] OR "Prostate neoplasm*"[tw] OR "Prostatic neoplasm*"[tw] OR "Prostate malignancy"[tw] OR "Prostatic malignanc*"[tw] OR "PCa"[tw]) AND ("Bone Neoplasms/secondary"[Mesh] OR "Lymphatic Metastasis"[Mesh] OR "Lymph node metasta*"[tw] OR "Lymphnode metasta*"[tw] OR "Lymphatic metasta*"[tw] OR "Bone metasta*"[tw] OR "Bony metasta*"[tw] OR "Osseous metasta*"[tw] OR "Metastasis bone" [tiab:~2] OR "Metastases bone" [tiab:~2] OR "Oligometasta*"[tw] OR "Oligo-metasta*"[tw] OR "Oligo metasta*"[tw] OR "mPCa" [tw] OR "metastatic prostate cancer" [tw])) AND ("Cytoreduction Surgical Procedures"[Mesh] OR "Prostatectomy"[Mesh] OR "Prostatic Neoplasms/surgery"[Mesh] OR "Cytoreductive prostatectom*"[tw] OR "Cytoreductive prostatectomy"[tiab:~2] OR "Radical prostatectomy*"[tw] OR "Local treatment"[tw]) NOT (animals [mh] NOT humans [mh])
